# Eight-year disease-free survival in clear cell adenocarcinoma of the urethra with surgery alone: a case report and literature review

**DOI:** 10.3389/fonc.2026.1841441

**Published:** 2026-07-09

**Authors:** Ji Wang, Yan Sun, Bo Jiang, Sichuan Hou, Zhijun Liu

**Affiliations:** 1 Department of Urology, Qingdao Municipal Hospital, Qingdao University, Qingdao, Shandong, China; 2 Anesthesia and Operation Department, Qingdao Municipal Hospital, Qingdao University, Qingdao, Shandong, China

**Keywords:** case report, long-term survival, minimally invasive surgery, urethral clear cell adenocarcinoma, urine cytology

## Abstract

We report a 39-year-old woman with clear cell adenocarcinoma of the urethra (CCAU). She was treated with surgery alone and has survived 8 years without recurrence. The patient had painless hematuria. Imaging showed a 5.1 cm urethral mass. The tumor invaded the bladder and vagina (clinical stage T3N2M0). Urine cytology confirmed the diagnosis, which is unusual for CCAU. She underwent laparoscopic radical cystectomy, lymph node dissection, and cutaneous ureterostomy. Pathology showed clear cell adenocarcinoma with CK7+/PAX8+. The final pathological stage was T3N0M0. She did not receive adjuvant therapy. Follow-up through January 2025 (8 years) showed no tumor recurrence, which appears to be rare among reported cases.

## Introduction

Clear cell adenocarcinoma of the urethra (CCAU) is a rare cancer. Only isolated case reports exist in the literature. Optimal diagnosis and treatment strategies are not well defined. CCAU is aggressive; reported 5-year survival rates are below 50% (1). We describe a 39-year-old female patient who presented with intermittent painless gross hematuria. Physical examination revealed a soft, mobile mass on the anterior wall of the mid-urethra. Laboratory tests including complete blood count, liver and kidney function were unremarkable. She was treated with surgery alone. This case was initially reported in 2019 (2). Here we present updated follow-up data. The patient has achieved 8-year recurrence-free survival.

## Case report

A 39-year-old Chinese woman was admitted on July 7, 2017. She was a non-smoker. She had intermittent painless gross hematuria for one month. The hematuria resolved spontaneously without any identifiable triggers. She had no urinary frequency, urgency, or dysuria. Vaginal examination revealed a soft, mobile mass on the anterior wall of the mid-urethra. The mass was about 5 cm in diameter. There was no tenderness. Laboratory tests were unremarkable, including complete blood count, liver function, kidney function, and coagulation profile.

**Figure 1:**
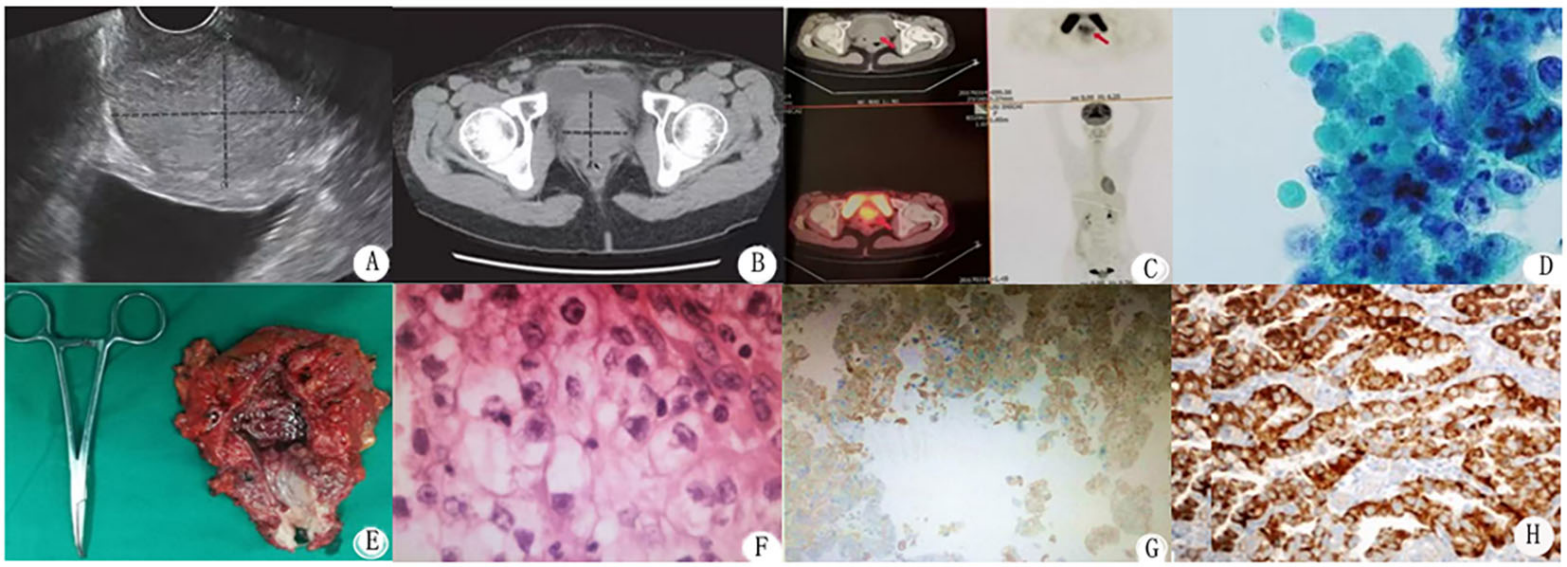
**(A)** Transvaginal ultrasonography revealed a round mass anterior to the vagina at the urethra. **(B)** CT examination showed irregular low density area of urethra. **(C)** PET-CT examination: perineal urethral mass, abnormal increase of glucose metabolism, consistent with malignant tumor manifestations. **(D)** Urine cytology examination revealed tumor cells exhibiting cellular enlargement with abundant clear to granular cytoplasm containing well-defined vacuoles, consistent with clear cell carcinoma. **(E)** The excised specimen. **(F–H)** The tumor cells exhibit vacuolization with abundant clear to granular cytoplasm and eosinophilic hyaline globules. The nuclei demonstrate vesicular chromatin and prominent nucleoli; Cytokeratin (CK) 7: Positive; PAX-8: Positive.

Transvaginal ultrasonography revealed a round mass anterior to the vagina at the urethra (**Figure 1A**). Contrast-enhanced computed tomography (CT) showed an irregular hypodense lesion within the urethra. The lesion measured 4.0 × 5.1 cm and protruded into the bladder lumen (**Figure 1B**). PET-CT revealed hypermetabolic activity in the perineal urethral mass. This finding was consistent with malignancy. The tumor involved the anterior vaginal wall. Bilateral inguinal lymphadenopathy was also noted (**Figure 1C**). Urine cytology confirmed clear cell carcinoma (**Figure 1D**). A preliminary diagnosis of urethral clear cell carcinoma was established. The clinical stage was T3N2M0.

After multidisciplinary consultation, the patient underwent surgery under general anesthesia. The procedure included laparoscopic radical cystourethrectomy, partial anterior vaginectomy, and extended pelvic lymphadenectomy. Bilateral inguinal lymphadenectomy was also performed. Intraoperatively, the tumor was localized to the posterior urethra near the bladder neck. It invaded the bladder neck and anterior vaginal wall. The excised specimen revealed a 4.0 × 3.9 × 2.5 cm³ mass at the vesicourethral junction (**Figure 1E**).

Histopathological examination confirmed clear cell adenocarcinoma. The cancer tissue did not involve the urethral margin, vaginal margin, or bilateral ureteral stumps. The bilateral pelvic, inguinal, and external iliac lymph nodes all showed reactive hyperplasia, with no evidence of tumor metastasis. The final pathological stage was T3N0M0. Immunohistochemical staining showed CK7(+), PAX8(+), CK20(−), P63(−), ER(−), and PR(−) (**Figures 1F–H**). No adjuvant therapy was administered after surgery.

## Follow-up

We followed the patient for eight years after surgery. CCAU has a poor prognosis. We advised the patient to have follow-up every three months. This included routine replacement of the ureteral stent. From the second to the fourth postoperative year, the patient had recurrent febrile episodes. These were all diagnosed as pyelonephritis and resolved after inpatient antibiotic therapy. In 2021, she was readmitted with fever. Contrast-enhanced CT showed several enlarged, fused lymph nodes in the left renal hilar region. The nodes measured approximately 2.1 cm × 1.0 cm. We recommended chemotherapy. The patient declined. She received antibiotic therapy and her fever resolved. No further treatment was given. Annual follow-up CT scans showed gradual reduction in the size of these lymph nodes. The most recent evaluation in January 2025 measured approximately 1.5 cm × 0.5 cm (**Table 1**).

**Table 1:** Follow-up data of the patient (2017–2025).

Date	Weight (kg)	Creatinine (μmol/L)	Urea nitrogen (mmol/L)	Max temp (°C)	eGFR (mL/min/1.73m^2^)	Left renal hilar enlarged LN size (cm^2^)
2017.3.20	46	62.39	3.54	–	>90.0	–
2017.7.21	46	72.24	4.44	–	91.0	–
2018.5.4	58	60.98	3.73	39	>90.0	–
2018.10.7	59	87.56	4.39	–	82.5	–
2021.4.15	52	83.5	5.3	–	86.3	2.1×1.0
2021.7.26	54	108.2	8.58	37.8	61.5	2.4×2.4
2022.1.12	51	113.3	8.22	–	58.3	2.4×1.0
2023.8.16	52	148.3	7.91	–	41.8	1.8×0.5
2024.2.22	52	144.6	7.75	–	43.2	1.5×0.5
2025.1.17	57	141.44	5.58	–	45.5	1.5×0.4

## Discussion

Female urethral malignancies are extremely rare, accounting for less than 1% of female urogenital tumors. Adenocarcinoma constitutes about 10% of these cases and is further subclassified into columnar, mucinous, and clear cell subtypes (1). Clear cell adenocarcinoma of the urethra (CCAU) mainly affects women, with a mean age of 58 years (range 35–80 years). CCAU lacks pathognomonic symptoms, and approximately 56% of cases are associated with urethral diverticula, which increases the difficulty of differential diagnosis (3). Cross-sectional imaging (CT/MRI) aids in lesion localization, but histopathological examination remains the diagnostic gold standard. Transurethral or incisional biopsy is typically required. In the present case, urine cytology unexpectedly identified malignant cells, suggesting that it may serve as a non-invasive diagnostic tool. The prognosis of CCAU is very poor. Yuvaraja B Thyavihally reported a single-center series of 18 cases, with a 5-year survival rate of only 33% (4).

Surgical resection is the main treatment for CCAU. Patel and colleagues showed that surgery improves overall survival and disease-specific survival (5). Radiotherapy and chemotherapy showed no survival benefit.

Preoperative imaging in our case showed enlarged lymph nodes. This raised concern for possible metastasis. We considered fine-needle aspiration biopsy to confirm nodal status. But we decided not to perform this procedure. A biopsy would not have changed our surgical plan. We wanted to minimize trauma and avoid delay in definitive treatment. Therefore, the patient proceeded directly to radical surgery with extended lymphadenectomy. Postoperative pathology confirmed no lymph node metastasis. PET-CT serves as a major modality for diagnosing malignant tumors and their metastases with a high positive diagnostic rate, yet false-positive findings still occur. As Tristan T. Demmert explained, this stems from elevated ^18^F-FDG uptake in inflammatory lesions owing to recruitment of abundant infiltrated and activated immune cells with high metabolic activity (6).

Before surgery, we talked with the patient at length about adjuvant radiotherapy and chemotherapy. We explained the possible benefits and risks. After careful discussion, she clearly refused any form of adjuvant therapy. Based on preoperative clinical staging, neoadjuvant therapy may delay the optimal timing of surgery. In addition, available studies lack sufficient evidence to confirm the clinical benefits of postoperative adjuvant therapy for patients. Therefore, adjuvant therapy was omitted in accordance with the patient’s preference.

After radical cystourethrectomy, we had several options for urinary reconstruction. The patient had advanced clinical stage (T3N2M0). We expected limited survival. Therefore, we chose cutaneous ureterostomy. This approach simplifies surgery. It avoids problems linked to more complex diversions. But it does affect quality of life. During eight years of follow-up, the patient needed to be hospitalized every three months to replace the ureteral stent to prevent stricture of the ureterocutaneous anastomosis. Even so, the patient developed hydronephrosis, bilateral renal atrophy, and slowly worsening kidney function (**Table 1**). Every type of urinary diversion after radical cystectomy can cause a gradual decline in renal function. These long-term problems match what Nishikawa and colleagues found. They followed 169 patients for 60 months after radical cystectomy with total urethrectomy. The cutaneous ureterostomy group had the highest rate of eGFR decline greater than 25% (7). Kassem S. Faraj evaluated renal function in 563 patients who underwent cystectomy with urinary diversion and reported that the mean 60-month GFR loss was 17% of baseline for the ileal conduit group and 14% for the orthotopic neobladder group (8). The exact reasons are not very clear but may include transient intraoperative ureteral clamping, postoperative ureteral anastomotic stricture or reflux, and postoperative pyelonephritis. In this case, the patient experienced recurrent postoperative fever, pyelonephritis, and transient ureteral dislodgement leading to hydronephrosis. These factors may all have contributed to the decline in renal function after surgery. Xiao-Dong Jin and colleagues suggested that urinary tract obstruction is the main cause of long-term renal impairment, and that patients with predisposing risk factors such as diabetes or hypertension are at increased risk of renal function decline (9). Long-term renal function monitoring is essential for patients with cutaneous ureterostomy.

## Conclusion

We report a female case of clear cell adenocarcinoma of the urethra. The patient was treated with surgery alone and had no recurrence during 8 years of follow-up. Preoperative diagnosis was confirmed by urine cytology, which provides a potential new approach for preoperative diagnosis of this rare tumor. Enlarged lymph nodes on imaging may represent reactive hyperplasia. Although no adjuvant therapy was given in this case, the decision for adjuvant treatment should be based on individual patient circumstances.

## Data Availability

The original contributions presented in the study are included in the article/supplementary material. Further inquiries can be directed to the corresponding authors.
